# A Possible Role for the Vagus Nerve in Physical and Mental Health

**DOI:** 10.3390/biom16010121

**Published:** 2026-01-12

**Authors:** Carola Y. Förster, Sergey Shityakov

**Affiliations:** 1 Department of Anaesthesiology, Intensive Care, Emergency and Pain Medicine, University of Würzburg, D-97080 Würzburg, Germany; 2 Texas Medical Research Network, Houston, TX 77074, USA; 3 Laboratory of Chemoinformatics, Infochemistry Scientific Center, ITMO University, 197101 Saint Petersburg, Russia; shityakoff@hotmail.com

**Keywords:** vagus nerve stimulation, transcutaneous VNS (tVNS), depression, inflammation, neuroimmunology, gut-brain axis, neuromodulation, treatment-resistant disorders

## Abstract

For decades, researchers have explored the therapeutic potential of the vagus nerve through vagus nerve stimulation (VNS). Initially developed for epilepsy, VNS has since been applied to treat resistant depression, stroke recovery, and inflammatory conditions. Transcutaneous VNS (tVNS) now offers a noninvasive alternative, fueling clinical trials in disorders ranging from rheumatoid arthritis and migraines to long COVID-19. Mechanistic studies suggest that afferent and efferent vagal fibers modulate immune responses, mood regulation, and neurotransmitter systems. The SPARC initiative has accelerated mapping of vagal circuits, enabling more precise approaches to stimulation. Despite progress, the results remain mixed: while some patients experience lasting symptom relief, others respond no better than to placebo. Depression studies, in particular, highlight both the promise and the complexity of VNS, as inflammation, motivation circuits, and gut–brain signaling emerge as key modulators. Next-generation closed-loop devices and circuit-specific targeting may improve efficacy and reduce adverse effects. VNS research thus lies at the intersection of neuromodulation, psychiatry, and immunology—offering hope for hard-to-treat conditions, yet demanding rigorous trials to separate myths from medicine. In this article, we review the current clinical and experimental applications of tVNS, analyze its mixed efficacy across psychiatric, immunological, and neurological disorders, and highlight the mechanistic insights, stimulation parameters, and emerging technologies that may shape next-generation therapies.

## 1. Introduction

**Figure 1:**
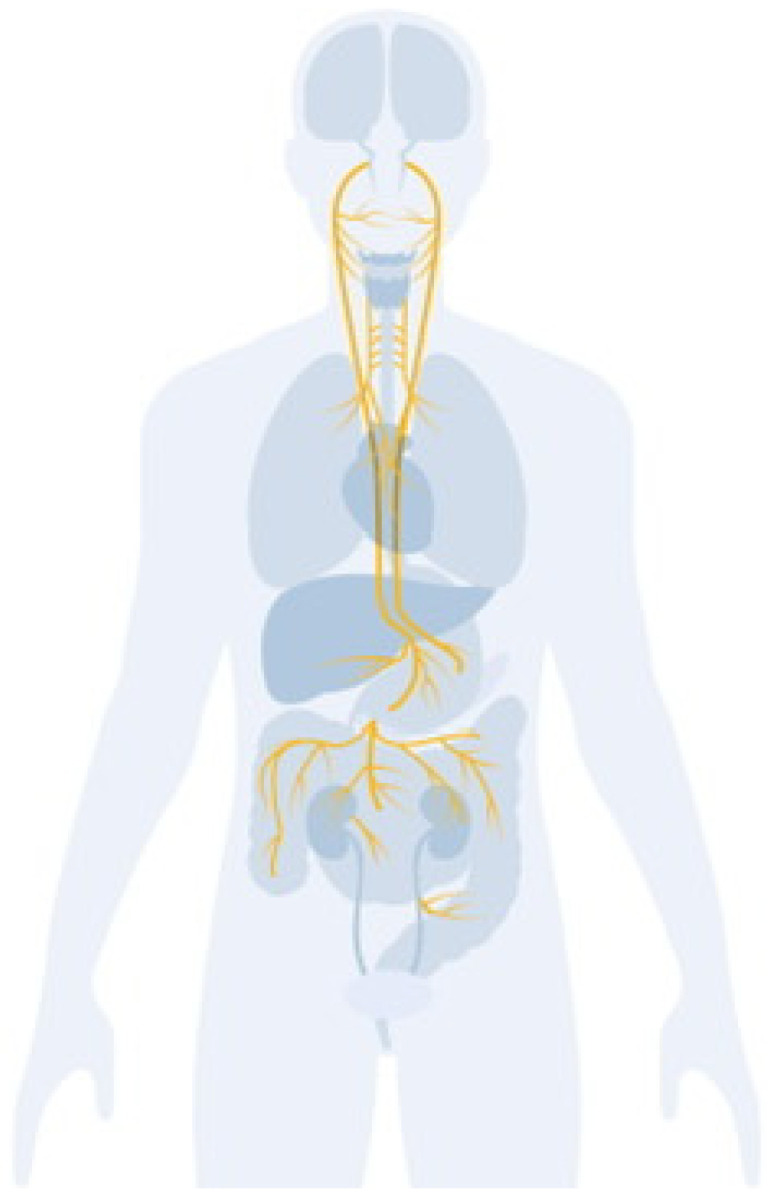
Anatomy of the vagus nerve and its branches. The illustration shows the course of the vagus nerve from the brainstem to thoracic and abdominal organs, highlighting its major branches and innervation targets in the heart, lungs, and gastrointestinal tract.

With roots in almost every organ and branch in the brain, the vagus nerve is a vine of nerve fibers [1] (Figure 1). It assists in identifying symptoms such as an overactive immune system, a racing heart, elevated blood pressure, discomfort, stomachaches, and even warning signals from gut microorganisms [2]. The vagus nerve, which runs from the brainstem into the abdomen, is the longest cranial nerve in the human body. The vagus nerve is widely distributed and is essential for controlling many physiological processes. Several organs are impacted by the vagus nerve, which serves as the parasympathetic branch’s primary channel. The VN helps control several bodily functions, including heart rate variability, gastrointestinal motility, and pancreatic function, by transmitting signals from the brain to these organs [1,3,4,5].

Within popular wellness narratives, the vagus nerve is frequently portrayed as a universal target for non-evidence-based interventions, purportedly capable of treating a wide range of conditions—from long COVID and headaches to cognitive decline, obesity, and mood disturbances [6]. Such claims are often overstated and lack rigorous empirical support. Nevertheless, a subset of research on vagus-nerve–mediated pathways and neuromodulation demonstrates mechanistic plausibility and clinically promising findings that justify continued scientific investigation.

## 2. Historical Background and Early Discoveries

Researchers have long recognized that delivering mild electrical pulses to the vagus nerve can help alleviate certain medical conditions. In 1997, the U.S. Food and Drug Administration (FDA) approved an implantable vagus nerve stimulation (VNS) device, surgically placed beneath the collarbone and connected via a lead wire coiled around the vagus nerve [7]. This device is primarily used for epilepsy patients who do not respond to conventional medications [3]. The FDA later cleared a similar VNS system in 2005 for the treatment of treatment-resistant depression [1,4], and in 2021, approved another for enhancing recovery after stroke [4].

**Figure 2:**
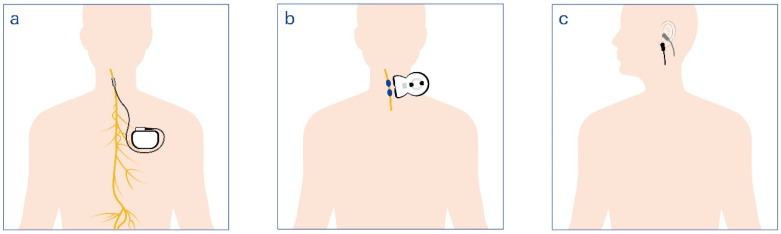
Schematic representation of invasive and noninvasive vagus nerve stimulation (VNS) approaches. (**a**,**b**): Conventional implanted (cervical) VNS, in which a pulse generator is surgically placed subcutaneously in the chest (**a**) and connected to an electrode cuff wrapped around the cervical branch of the vagus nerve (**b**). This system delivers intermittent electrical impulses to modulate afferent and efferent vagal fibers. (**c**): Transcutaneous VNS (tVNS) techniques, which deliver stimulation through the skin without surgical implantation. The cervical tVNS approach targets the vagus nerve in the neck via surface electrodes, whereas the auricular tVNS approach targets the auricular branch of the vagus nerve at the outer ear (typically the tragus or cymba conchae). Both methods aim to activate similar neural pathways involved in autonomic and inflammatory regulation, although with different depths and selectivities of stimulation.

In addition to implanted devices, non-invasive vagus nerve stimulators—applied externally to areas such as the outer ear or neck—have gained regulatory approval in the United States and many other countries for managing conditions including obesity, chronic pain, and migraines [5,8] (Figure 2).

In 2015, the National Institutes of Health (NIH) Common Fund launched a $250 million initiative to explore the therapeutic potential of vagus nerve stimulation (VNS), with a second phase scheduled to begin in 2022—reflecting strong confidence in this approach. This effort is part of the SPARC (Stimulating Peripheral Activity to Relieve Conditions) program [9,10], which seeks to precisely map the specific nerve fibers and neural circuits involved in peripheral nerve signaling and clarify how they influence bodily functions.

Researchers anticipate that insights from SPARC will not only refine existing treatments but also spur the development of novel therapies for a range of conditions, including inflammatory bowel disease and long COVID-19 [11,12]. Currently, clinical trials are underway for transcutaneous VNS (tVNS) devices—non-invasive systems that stimulate the vagus nerve through the skin. These devices offer greater accessibility and ease of use compared to implanted counterparts and have demonstrated encouraging results in early studies for treating rheumatoid arthritis, migraine, lupus, and chronic fatigue syndrome [1,13,14] (Figure 2).

The adoption of fundamentally new biomedical concepts is often a slow process, frequently requiring several decades before becoming broadly accepted within the scientific and clinical communities. As noted in analyses of translational research dynamics, transformative ideas may take twenty to forty years to achieve widespread recognition and implementation in practice (Tracey, K. J., Feinstein Institutes for Medical Research, Manhasset, NY). The notion of the so-called “vagus vine” remains partly embedded in anecdotal interpretation, and the available evidence is still inconclusive. Nevertheless, a growing number of researchers consider vagus nerve-based modulation as a promising avenue for patients suffering from otherwise difficult-to-treat conditions [1].

The vagus, which means “wandering” in Latin, is the name given to the longest of the brain’s nerves by English neuroanatomist Thomas Willis in 1664.

According to Tracey, “there are actually two, one on each side of your body, but we call it the vagus nerve, singular.” Up to 100,000 fibers can be found on each side, and each fiber supports a distinct function, such as speaking, breathing, immunity, heart rate, or contractions of the gut [15], that aid in food digestion.

The majority of vagal nerve fibers are afferent (ca. 80%), reporting to the brain about the body’s condition, whereas only 20% are efferent, conveying instructions from the brain [1].

In the late 19th century, the British scientist Walter Holbrook Gaskell reported that efferent signals often regulate and calm visceral function, whereas afferent signals may increase arousal and responsiveness [16]. In the 1880s, American neurologist James Leonard Corning was the first to stimulate the vagus nerve with electricity—using a vibrating fork-like instrument on the neck in an attempt to reduce cerebral blood flow and treat epilepsy [17].

While unsuccessful, his approach foreshadowed later breakthroughs. A century later, the neurologist Jacob Zabara at Temple University discovered that vagal nerve stimulation (VNS) could reduce seizures in dogs by modulating excitability in central circuits [18].

This pivotal insight led to the first implantation of a human VNS device for epilepsy in 1988 by the neurosurgeon William Bell and neurologist James Kiffin Penry—a milestone in modern neurotherapeutics [19]. Thus, a warning signal sent down by the vagus nerve signals the brain to soothe inflammation in the body [6].

## 3. Mechanistic Insights and Serendipitous Discoveries

The vagus nerve comprises approximately 80% afferent fibers, which convey sensory information from peripheral organs to the central nervous system, and 20% efferent fibers, which transmit regulatory signals from the brain to the body [20]. Afferent pathways primarily sense visceral states, such as inflammation or gut signals, and project to brainstem nuclei like the nucleus tractus solitarius, influencing higher brain regions involved in emotion and homeostasis. In contrast, efferent pathways, part of the parasympathetic system, modulate peripheral functions like heart rate and digestion via acetylcholine release [20].

In the inflammatory reflex, afferent vagal fibers detect peripheral cytokines, relaying signals to brainstem nuclei to trigger central anti-inflammatory responses [21]. However, in rats no vagal efferents synapse activating splenic sympathetic fibers was determined in the electrophysiological experiments [22].

VNS devices, which deliver pulses every few minutes, are direct descendants of Zabara’s invention. According to a pivotal study, it reduced seizures by 45% on average after a year. The mechanism of action is believed to be based primarily on the stimulation of afferent fibers, which leads to the brain [23].

The medication produced a striking side effect: it gradually made patients happier, even though their seizures persisted. As Kevin Tracey noted, when doctors later offered to remove the implanted device, many patients declined, saying, “No, leave it in, it makes me feel good.” These anecdotal observations prompted the manufacturer to market its vagus nerve stimulator as a novel therapy for depression [19]. This serendipitous finding ignited a surge of research, still far from cresting, aimed at clarifying the vagus nerve’s precise role in mood regulation [24]. Scientists now understand that key brain regions implicated in mental illness, including the amygdala (which processes emotions like fear, anxiety, and stress), the hypothalamus (which governs the release of stress hormones such as cortisol), and the ventral tegmental area (central to motivation, reward, and pleasure), all receive constant input from the body via the vagus nerve [25,26]. These afferent signals—relaying information about digestion, heart rate, and overall physiological status—first arrive at the nucleus tractus solitarius, the brainstem’s primary integration hub, which sorts and distributes them to higher brain centers [27]. In addition to carrying signals upward to the brain, the vagus nerve also sends descending signals that help regulate internal functions like heart rate, thereby maintaining the body’s internal balance, or homeostasis [28].

“Fight-or-flight” hormones increase blood pressure and heart rate in response to danger while reducing intestinal and gastrointestinal activity. The vagus nerve provides real-time feedback by detecting these changes and communicating them to the brain. It also makes fine-tuning easier. The brain triggers the opposing “rest-and-digest” mechanism by sending signals down the vagus nerve when stress signals become too strong. At the tips of its roots, the wine releases the neurotransmitter acetylcholine, which improves digestion in the stomach and lowers blood pressure and heart rate in the heart [29]. The system becomes more relaxed.

In the late 1990s, a second coincidental discovery revealed that the vagus nerve is capable of much more than just calming the body. In Tracey’s laboratory, researchers investigated a medication that decreases inflammation in the brain. Certain types of inflammation, such as redness and swelling surrounding a wasp bite, are protective because they indicate that the immune system is coping with the venom. However, tissues can be harmed by inflammation caused by an overactive immune system. Pro-inflammatory cytokines are signaling chemicals released into the circulation by splenic cells in response to injury or infection, which triggers the immune response at the location [30]. Insidious injury can result from inflammation if cytokines are constantly present for months or years due to stress, a chronic infection, or an autoimmune condition. Researchers have given mice an injection of a poison that causes an immunological response to evaluate their anti-inflammatory effects [31].

The anti-inflammatory medication they later administered to the brain decreased the body’s degree of inflammation. How did that occur? The medication should not have been able to leave the brain because of the blood-brain barrier [32]. Tracey remembers, “We literally talked about this for months.” The drug signal is sent from the brain deep into the body by the vagus nerve [33] in individuals with major depression. The authors detailed that animals achieve internal homeostasis by balancing pro-inflammatory and anti-inflammatory pathways during infection or injury. The endotoxin (lipopolysaccharide) produced by all Gram-negative bacteria activates macrophages to release cytokines that are potentially lethal. Inflammatory responses to endotoxin are regulated by the central nervous system through humoral mechanisms. Afferent vagus nerve fibers activated by endotoxins or cytokines stimulate hypothalamic–pituitary–adrenal anti-inflammatory responses [32].

**Figure 3:**
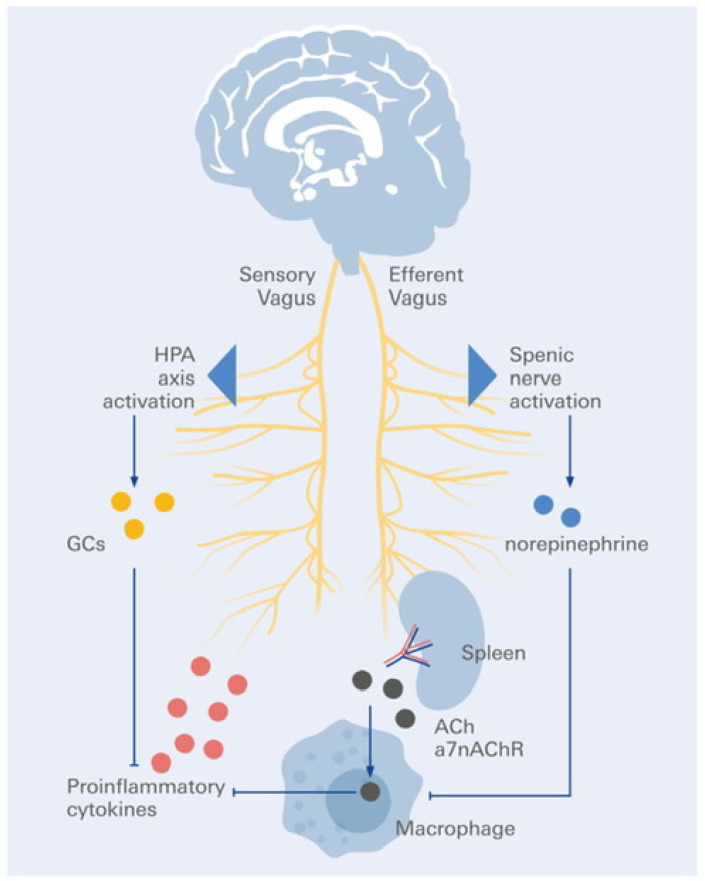
Anti-inflammatory splenic pathway of vagus nerve activation. Activation of the vagus nerve triggers an anti-inflammatory reflex via efferent signaling to the spleen. Through the splenic nerve, acetylcholine (ACh) is released from cholinergic T cells and binds to α7 nicotinic acetylcholine receptors (α7nAChR) on macrophages, thereby inhibiting pro-inflammatory cytokine release (e.g., TNF-α, IL-1β, IL-6). This cholinergic anti-inflammatory pathway links neural activity to immune regulation and systemic inflammation control.

Moreover, Tracey reported that using electricity to activate the vagus nerve also reduced inflammation throughout the body without the need for medication [33]. He remembers it as a “life-changing” moment. Inflammation is linked to approximately half of all disease-related deaths, including those from diabetes, asthma, heart disease, stroke, autoimmune diseases, and neurodegenerative diseases [34,35,36] (Figure 3).

## 4. Clinical Evidence: VNS in Depression

Starting with depression felt like a smart idea. Although the symptoms of depression vary from person to person, melancholy, a lack of motivation, and social disengagement are some of the prevalent symptoms. Inflammation is also present in nearly one-third of individuals with severe depression [37]. “Depression is caused by cytokines,” claims Tracey. “You will become exhausted and lose interest in things that typically make you happy if I inject you with these inflammatory molecules” [38]. He added that physicians frequently prescribe prophylactic antidepressants to cancer patients undergoing treatment that involves cytokines [39,40]. Indeed, targeting pro-inflammatory markers, including C-reactive protein and cytokines, shows promise as a potential therapeutic approach for subgroups of patients with fear- and anxiety-based disorders [41].

Vagus nerve stimulation (VNS) was initially heralded as a promising therapy for depression, but its momentum slowed shortly after gaining traction. The U.S. Food and Drug Administration (FDA) approved VNS for treatment-resistant depression in 2005, based on clinical studies showing that it alleviated symptoms in at least 30% of patients within one year of use [42]. However, two years later, the Centers for Medicare & Medicaid Services (CMS) declined to cover the treatment, citing insufficient evidence of its effectiveness (CMS, 2007, Decision Memo for Vagus Nerve Stimulation for Treatment of Resistant Depression [CAG-00313R]). With a price tag of $30,000 or more in the United States, VNS became financially inaccessible for most patients. Support for its efficacy grew nonetheless: a 2017 study of 800 individuals with treatment-resistant depression found that after five years of VNS therapy, 43.3% achieved remission and 67.6% experienced at least a 50% reduction in symptoms [1].

Now, a large-scale clinical trial, optimistically named RECOVER [43], aims to provide the robust evidence needed to secure Medicare coverage. The study enrolled 1000 patients with severe major depressive disorder who, on average, had failed to respond to 13 prior treatments and had a history of suicide attempts—characteristics that typically exclude individuals from standard pharmacological trials [44].

For instance, in the ongoing RECOVER trial, some participants with decades of failed therapies report sustained benefits from implanted VNS. A participant in the RECOVER clinical trial, initiated in the summer of 2021, received an implanted vagus nerve stimulator positioned subcutaneously near the clavicle. The device delivers intermittent electrical impulses of approximately two milliamperes through a lead coiled around the cervical branch of the vagus nerve adjacent to the laryngeal structures. The stimulation can transiently affect vocal tone and phonation, which serves as an indirect indicator of proper device function [45].

Prior to implantation, the patient had undergone multiple conventional treatments for major depressive disorder over a period of three decades, including psychotherapy, pharmacotherapy, transcranial magnetic stimulation, and electroconvulsive therapy, all of which produced only transient improvements [46]. Over the months following device activation, gradual behavioral and affective changes, including a restoration of emotional responsiveness and the affective range, were noted. Approximately two years after stimulation began, antidepressant medication was discontinued under clinical supervision, as the patient reported stable mood regulation attributed to the neuromodulatory intervention.

## 5. Inflammation, Neuroimmune Interfaces, and VNS

VNS produces therapeutic effects through an integrated neuro-immunomodulatory framework [1]. By enhancing monoaminergic and dopaminergic neurotransmission, upregulating BDNF-mediated neuroplasticity, and suppressing systemic inflammation via the cholinergic anti-inflammatory pathway, VNS concurrently addresses the core dysregulations observed in affective, inflammatory, and neurological disorders, providing a unified mechanistic explanation for its multimodal efficacy.

However, in June 2024, interim findings from the RECOVER trial—based on data from more than 500 participants—revealed mixed results. While many patients with depression who received active vagus nerve stimulation showed meaningful improvement, a comparable level of benefit was also observed in those whose devices were not activated (During the first year of the trial, participants were blinded to whether their device was delivering stimulation, though one participant, Bolton, reported she could still sense when it was active). The results underscore the powerful—and still not fully understood—role of the placebo effect in depression treatment [47]. Dr. Sarah Lisanby, director of the National Institute of Mental Health’s Division of Translational Research, described the outcome as “sad but not surprising.”

She reported that the placebo response interferes with all investigations of psychiatric equipment [48]. Furthermore, she noted that research on VNS is limited in comparison to the decades of evidence supporting electroconvulsive therapy, which relieves depression in up to three-quarters of patients but impairs memory (UK ECT Review Group, 2003) [49] (among other side effects that Bolton found unacceptable).

The RECOVER study is still ongoing in the interim. The data, according to Conway and other researchers, should be able to predict who would most likely gain from further VNS studies. Although inflammation was not monitored in this study, it may be a crucial indicator. A pilot study on individuals with depression who had elevated inflammatory markers was published in February 2024 by researchers at the University of Montreal. Nearly all of them showed notable improvement, as their inflammation subsided four years after VNS [42].

Moreover, researchers, such as Andrew Miller of Emory University and Charles Raison of the University of Wisconsin–Madison, have discovered ways in which inflammation might lead to depression. The protective barrier between blood vessels and the brain can be weakened or even disrupted by inflammatory cytokines that are present in the blood [50,51]. Once within the brain, they cause the production of more inflammatory chemicals by immune cells known as microglia. Inflammation in the brain can disrupt the synthesis of neurotransmitters, such as dopamine and serotonin, which decreases motivation, pleasure, and feelings of well-being. Additionally, it decreases the synthesis of BDNF, a substance that promotes neuronal growth and connectivity. Neuronal connections deteriorate as BDNF levels decrease. This makes it more difficult for the hippocampus, which is involved in learning and memory, to recover from a stressful incident, and for the prefrontal cortex, which is the part of the brain that helps us control our emotions, to suppress alarm signals from the amygdala. Could this depressive cycle be disrupted by the vagus nerve, reducing inflammation in the body? Its anti-inflammatory channels and their mechanisms of action have been mapped by Tracey and colleagues. In response to inflammation detected by vagal afferents, the brain activates a neural circuit via the splenic sympathetic nerve and suppresses inflammatory cytokine production. The spleen, which houses immune cells, releases acetylcholine in response to these commands. Acetylcholine causes macrophages, which are white blood cells, to produce fewer pro-inflammatory cytokines [35].

Additionally, it might trigger the normal function of splenic macrophages to kill damaged or infected tissues to assist in tissue regeneration by traveling to sites of inflammation, such as the gut. According to Tracey, macrophages may even be able to repair the harm that inflammation does to the brain and stimulate the development of new neurons and circuits in their restorative reincarnation: Small amounts of inflammatory chemicals generated by injured tissues have been shown in experiments to trigger afferent signals via ascending vagus nerve fibers, which serve as the sensory arm of a “inflammatory reflex.” Acetylcholine is quickly released in organs as a result of the following activation of vagal efferent fibers, which are the motor arm of the inflammatory reflex. On the other hand, a disturbance in the impulses that flow down the vagus nerve can prevent the nerve’s anti-inflammatory role from being activated in inflammatory illnesses, such as depression, as Tracey suggested. Inflammation can become chronic and detrimental if the pathway is disrupted or if the signal is too faint [52].

However, turning all of this knowledge into medicines has proven challenging. VNS does not always reduce inflammation, according to a recent meta-analysis headed by Sharmili Edwin Thanarajah of the University Hospital Frankfurt in Germany.

A PRISMA-compliant meta-analysis of 36 human studies (780 VNS) reported no significant cytokine reductions (TNF-α, IL-6) after short- or long-term VNS, with small, non-significant effect sizes [53]. Significant CRP decreases occurred only in a subgroup of 4 long-term studies with acute inflammation. Mixed responses appeared in populations like RA and Crohn’s, limited by low per-disease study numbers. Implanted VNS showed stronger trends than tVNS, but parameters, immune challenges, and quality (mostly poor–fair) were non-significant moderators.

Additionally, VNS may decrease depression but not inflammation in third-year depressed individuals whose blood contains proinflammatory cytokines. This is also suspected by the authors of [54].

Depression is a complicated and multifaceted illness. According to Tracey, “while depressed people may share a similar appearance, they may not all suffer from the same illness.” Because of this variability, different vagus nerve signal patterns may work better for different people. Some people may benefit more from signals that travel from the brain to reduce inflammation and calm the body, whereas others may benefit more from signals that travel down.

## 6. Neuroimaging and Neurochemical Correlations

Neuroimaging provides hints. In general, vagus stimulation improves connections between the prefrontal cortex and the amygdala, which may result in improved emotional regulation, although the results vary depending on the type of VNS and the regimen utilized. Additionally, it increases activity in the left anterior insula, a region linked to emotion processing. Additionally, a group headed by Jian Kong from Harvard Medical School and Massachusetts General Hospital discovered that when VNS is used to treat depression, it seems to improve connectivity between the rostral anterior cingulate cortex, which is linked to self-referential thinking, and the medial hypothalamus, which controls stress responses. This change can be a sign of greater emotional and cognitive integration [55].

Resting-state functional MRI (fMRI) studies in patients with depression have shown that VNS increases functional connectivity within the amygdala-lateral prefrontal network, a change associated with improved emotional regulation [56,57].

A VNS-induced increase in the neurotransmitters serotonin and norepinephrine, which have been linked to increased energy and alertness in mouse/rat studies, may account for some of these benefits. Additionally, research on animals has shown that VNS increases BDNF, which aids in reestablishing neuronal connections damaged by stress and depression [58].

Additionally, this therapy seems to restore other signaling chemicals, such as glutamate and gamma-aminobutyric acid, which are commonly out of balance in depression.

**Figure 4:**
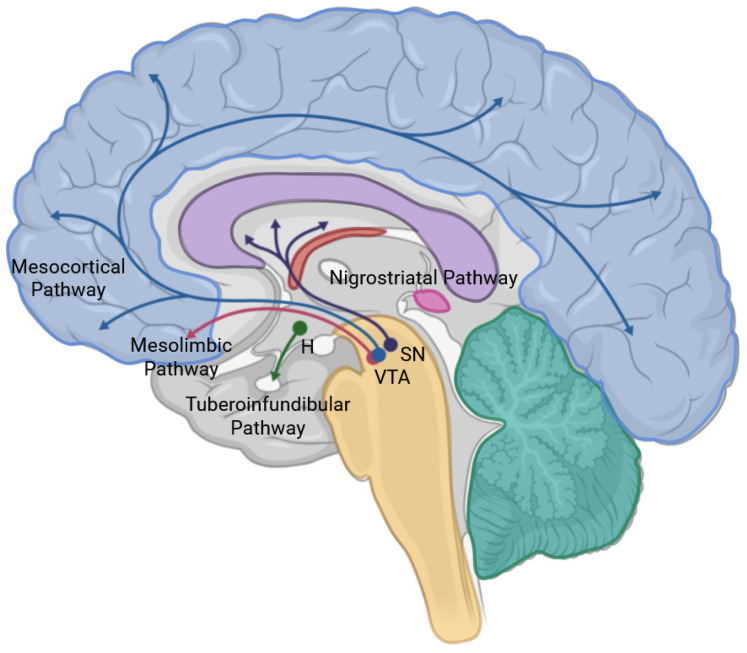
Schematic of the key dopaminergic pathways and the potential improvement of dopamine circuits via VNS. The illustration depicts projections originating from the substantia nigra (SN) and ventral tegmental area (VTA). The nigrostriatal pathway (purple) projects from the SN to the striatum. The mesocortical (blue) and mesolimbic (pink) pathways project from the VTA to the cortex and limbic structures, respectively. The tuberoinfundibular pathway (green) from the hypothalamus to the pituitary regulates prolactin release.

However, one of the more convincing theories is the impact of VNS on dopamine circuits. People who are depressed have low levels of dopamine, a neurotransmitter that is essential for motivation and pleasure. Conway and colleagues employed imaging more than ten years ago to examine the effects of a year of VNS on the brains of individuals suffering from serious depression. They discovered that the ventral tegmental region, which produces dopamine, was more activated in patients who responded to treatment [59]. Additionally, several recent studies [60] suggest that VNS can improve dopamine circuits in the brains of major depressive disorder patients and in vivo models (Figure 4).

In a 2024 study, the neuroscientist Nils B. Kroemer, who works at the Universities of Bonn and Tübingen in Germany, administered tVNS to depressed patients, as they repeatedly pressed a button to raise a ball in exchange for tiny rewards. They are greatly energized by tVNS, which also increases their motivation to obtain food and money [61].

A game and an hour-long tVNS session address only the lack of motivation and desire, which is a symptom of serious depression. However, any progress is good for an illness that can be so crippling. According to Kroemer, decreased sensory input to the brain may be the cause of low motivation in at least some depressed individuals. We obtain a sensation of drive—a hunger, both literally and figuratively—from internal impulses from the gut and other organs that travel through the vagus nerve. “There seems to be a strong hardwired motivational signal that allows us to explore new options if the stomach is empty,” Kroemer says. However, it only occurs if the messages are transmitted, which necessitates a functional vagus nerve.

## 7. Vagal Pathways Linking Gut Microbiota to Motivation and Mood: Implications for taVNS

Researchers such as Kroemer have investigated how the gut microbiota affects motivation and how it interacts with tVNS. Signals from gut bacteria and their byproducts travel up the vagus nerve to the brain and the nucleus tractus solitarius. These pathways alter the release of neurotransmitters that control mental states, such as serotonin and dopamine. Additionally, the brain communicates with the stomach through the vagus nerve, affecting digestion and inflammation, two characteristics of the gut environment that in turn impact the makeup of the bacteria that live there. While pathogenic germs may exacerbate depression, anxiety, panic attacks, and stress, there is evidence that good bacteria might decrease these conditions [62]. VNS is only available for a small number of patients with depression or other mental illnesses outside of clinical trials (approximately 125,000 persons have received an implant). Instead, because tVNS is more convenient and less expensive, more researchers and physicians are using it. According to Conway, “because it is linked to the nerve and delivers a signal 24/7 for certain,” a surgically implanted device is assumed to be more effective. Additionally, implants stimulate more brain regions than tVNS does. According to imaging studies, other drawbacks of externally applied VNS include the fact that devices that attach to the ear mainly stimulate afferent fibers, whereas those applied to the neck might not effectively reach the deep-seated vagus nerve.

The majority of tVNS research has been modest and constrained. According to a randomized study headed by Kong, tVNS given through the ear for eight weeks was just as successful in treating major depression as the antidepressant citalopram (Celexa) [63].

The FDA granted the treatment a “breakthrough device” designation, expediting its development and regulatory review, following a 2021 pilot study led by Omer T. Inan of the Georgia Institute of Technology and J. Douglas Bremner of Emory University. That study found that three months of twice-daily transcutaneous vagus nerve stimulation (tVNS), self-administered to the neck, reduced stress symptoms by 31% compared with a control group and blunted participants’ inflammatory response to traumatic memories, a finding consistent with earlier research on tVNS effects on PTSD symptoms and interleukin-6 levels [1]. Supporting this, another small pilot study at Leiden University in the Netherlands showed that individuals classified as “high worriers” experienced fewer intrusive thoughts after receiving ear-clip tVNS compared to those receiving sham stimulation, as noted in preliminary tVNS literature (e.g., Koenig et al. [64]).

Taken together, most mechanistic insights into microbiome–vagus interactions originate from rodent studies, where invasive methods demonstrate that microbial metabolites can activate vagal afferents, alter neurotransmitter release, and modulate anxiety- and motivation-related behaviors. Human evidence, by contrast, derives mainly from observational cohort studies and non-invasive stimulation experiments showing associations between gut microbial composition, inflammatory markers, and mood or stress responses, but without direct proof of vagal causality. Several proposed mechanisms—such as specific bacterial strains driving vagal signaling or tVNS reliably modifying microbiota profiles—remain preliminary, supported only by small pilot trials or indirect physiological markers.

## 8. Technological Advances and Future Directions

Clinicians are increasingly integrating tVNS with traditional therapies such as cognitive-behavioral therapy and medications. Additionally, these devices allow people to self-treat a wide range of ailments, such as those associated with stress, anxiety, and even general malaise. However, there is no universally accepted methodology for any particular situation; much worse, the inability to target particular fibers may result in unintended consequences. VNS has more than only relaxing effects, unlike what many people think. Some pathways activate arousal, promoting alertness and vigor—or, if overstimulated, jitteriness and worry.

Moreover, SPARC researchers have compiled a massive data-sharing platform that includes detailed maps and models of the vagus nerve, along with other tools, with new submissions being continuously integrated.

**Figure 5:**
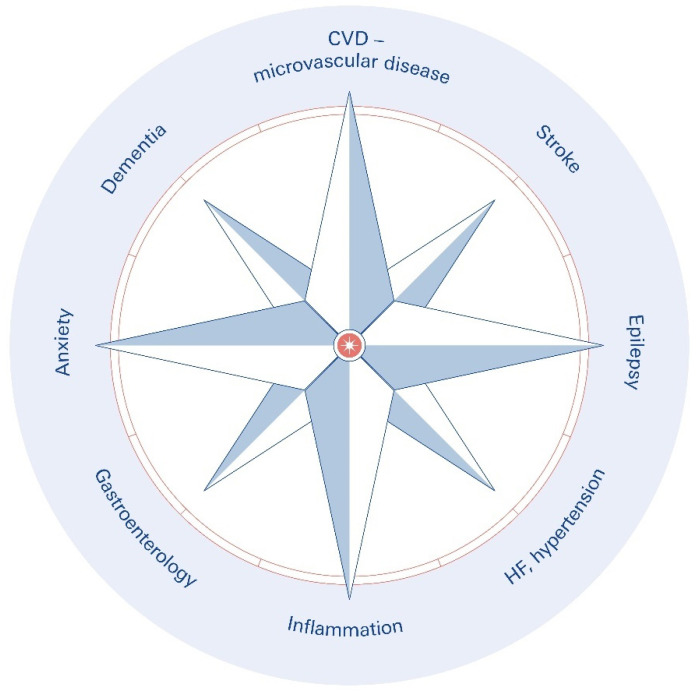
Overview of the clinical and mechanistic applications of vagus nerve stimulation (VNS) and transcutaneous VNS (tVNS), as discussed in the manuscript. The focus is on neurological disorders, psychiatric disorders, immunological and inflammatory disorders, cardiovascular disorders, gastrointestinal and metabolic aspects, and cross-cutting mechanisms and technologies.

SPARC teams seek to separate individual fibers and circuits, as well as their pathways, and monitor their activities by utilizing artificial intelligence and other technologies. The objective is to create methods for focusing on certain nerve fibers implicated in a range of disorders. Pain management, traumatic brain injury, Parkinson’s illness, Crohn’s disease, atrial fibrillation [50] and type 2 diabetes mellitus are all on the ambitious list [65,66] (Figure 5).

These technologies may soon be more customized. Activating several connections along the vagus nerve to stimulate fibers associated with particular organs while avoiding those that have negative consequences is one of the ongoing breakthroughs in VNS. Moreover, enhanced transcutaneous electrical nerve stimulation (eTENS) already demonstrates improved nerve-fiber recruitment profiles in preclinical models [67], yet fiber-specific targeting remains experimental and is not yet available in clinical VNS systems.

Scientists may be able to modify stimulation levels via newly developed “closed-loop” devices that react to inputs from the body in real time, such as inflammation, heart rate, or food desires [68,69].

According to some supporters, VNS can take completely other shapes. Once a neuronal circuit has been located, it can be targeted via a variety of techniques, such as targeted ultrasonography or microscopic implants in different body areas, including the brainstem. Researchers at Columbia University’s Zuckerman Institute discovered in 2024 the exact circuit in the vagus nerve and nucleus tractus solitarius that alerts the brain to new inflammation in the body and controls the reaction—basically, the dial for inflammation—which they suggested treating with medication [70].

Amidst these highly technical advances, individual experiences can highlight the human dimension: one patient described how, months into treatment, she suddenly found herself tapping to the rhythm of the radio in sync with her device’s pulses—and even singing again after years of silence. For her, VNS marked the moment she felt that life was returning.

## 9. Conclusions

Vagus nerve stimulation has evolved from serendipitous epilepsy treatment to promising, although still incompletely understood, therapies for inflammatory and affective disorders. Its bidirectional neuroimmune and neurovisceral influence places it at the crossroads of psychiatry, neurology, and immunology. Future research should refine mechanistic specificity, optimize stimulation parameters, and clarify biomarkers of response to harness the full therapeutic potential of the “vagus vine”.

Key mechanistic insights reveal its role in modulating limbic connectivity, enhancing monoaminergic and dopaminergic transmission, upregulating BDNF-mediated plasticity, and suppressing systemic inflammation via the cholinergic anti-inflammatory pathway. However, significant limitations remain, including heterogeneous clinical responses, a lack of reliable biomarkers for patient stratification, inconsistent anti-inflammatory effects in human studies, and the methodological challenge of distinguishing afferent from efferent mechanisms in non-selective stimulation. Future priorities should focus on biomarker-driven patient stratification, the refinement of stimulation parameters through closed-loop systems, the development of advanced fiber-selective targeting technologies, multimodal mechanistic studies integrating neuroimaging and immunophenotyping, and controlled longitudinal trials that account for placebo effects. By addressing these priorities, VNS can advance from a broadly applied intervention toward a precision neuromodulatory tool tailored to individual pathophysiology.

## Data Availability

No new data were created or analyzed in this study.

## Abbreviations

The following abbreviations are used in this manuscript:

VNS	Vagus Nerve Stimulation
tVNS	Transcutaneous Vagus Nerve Stimulation
FDA	Food and Drug Administration
SPARC	Stimulating Peripheral Activity to Relieve Conditions
CMS	Centers for Medicare and Medicaid Services
BDNF	Brain-Derived Neurotrophic Factor
PTSD	Post-Traumatic Stress Disorder
